# Biocontrol potential of hive-associated *Bacillus* species against American Foulbrood disease in honeybees (*Apis mellifera* L.)

**DOI:** 10.3389/finsc.2026.1870750

**Published:** 2026-10-08

**Authors:** Anuja Shrestha, Yva Eline, Kenzie Sundstrom, James T. Van Leuven

**Affiliations:** 1 Department of Animal, Veterinary and Food Science, University of Idaho, Moscow, ID, United States; 2 Bioinformatics and Computational Biology Program, University of Idaho, Moscow, ID, United States

**Keywords:** *Bacillus*, *Paenibacillus larvae*, *Apis mellifera*, bee pathogen, bee larvae, biocontrol, gut microbiome

## Abstract

**Introduction:**

Honeybee (*Apis mellifera* L.) larvae are susceptible to *Paenibacillus larvae*, a spore-forming, Gram-positive bacterium that causes American Foulbrood (AFB), a destructive honeybee brood diseases. Current antibiotic treatments are inadequate because of rising pathogen resistance and residue accumulation in hive products, driving interest in biological and other natural strategies for AFB control. Most candidate biocontrol agents, however, have been evaluated only for their ability to inhibit *P. larvae* or protect larvae, with less attention to their effects on adult bees.

**Methods:**

Here we assessed the biocontrol potential of hive-associated *Bacillus* species against *P. larvae* infection. Bacteria isolated from AFB-infected combs were sequenced and screened for antagonistic activity, and active isolates, their lysates, and lysate-derived protein fractions were tested for their ability to improve larval survival during *P. larvae* challenge. To evaluate effects on adult bees, newly emerged workers were caged and fed the *B. licheniformis* lysate (ASx), and their survival and gut microbiome were compared with untreated controls and with bees fed Terra-Pro, a Terramycin-based AFB treatment.

**Results:**

Several *Bacillus* species – including *B. zhangzhouensis, B. subtilis, B. amyloliquefaciens, B. licheniformis*, and *B. mojavensis* – inhibited one or more *P. larvae strains*. The *B. licheniformis* lysate (ASx) and its 30 -50 kDa protein fraction improved larval survival during *P. larvae* challenge. In caged adults, survival of lysate-fed bees differed from untreated controls but was comparable to that of Terra-Pro-fed bees, and their gut microbiome remained similar to that of untreated controls.

**Discussion:**

Together, these results indicate that hive-associated *Bacillus* species – and *B. licheniformis*-derived proteins in particular – are promising candidates for AFB management that improve larval survival without harming adult bees more than an existing antibiotic treatment.

## 1 Introduction

Managed honeybees (*Apis mellifera* L.), one of the most important crop pollinator species, provide around 50% of global crop pollination (1) and play a significant role in global food production, contributing approximately one-third of the total human food supply (2). In the United States, managed honeybees alone add $15 billion worth of crop pollination services each year (3, 4). However, beekeepers have consistently experienced high losses of managed honeybee colonies each year due to a plethora of biotic and abiotic risk factors (5). One honeybee illness that is particularly devastating is American foulbrood disease (AFB).

AFB is an economically important bacterial disease in honeybee larvae that has spread globally. It is caused by *Paenibacillus larvae* (*P. larvae*), a spore-forming, Gram-positive, highly specialized pathogen with only one established host, honeybee larvae (6, 7). The bacterium infects young larvae via the ingestion of contaminated food, after which the bacterial spores germinate, and the vegetative bacteria propagate rapidly and proliferate in the midgut before they breach the epithelium and invade the hemocoel (8, 9), causing larval death. The dead larvae degrade and contain millions of spores readily transmissible between colonies by foraging bees or apiculturists (10). These spores are highly heat-resistant and stable for many years, making the control of AFB very difficult (10).

AFB has been particularly devastating because of inadequate current treatment options. Burning colonies and contaminated hive materials are expensive for beekeepers, resulting in the loss of both hive materials and productive hives. Alternatively, the use of antibiotics, such as oxytetracycline or tylosin, to clear the infection is commonly practiced in some countries. However, treatments are not sufficient to combat the disease due to increased pathogen resistance and the accumulation of antibiotic residuals in honeybee products (11–13). Additionally, antibiotics are not permitted for use in European countries, leaving only the physical elimination of infected colonies for mitigation options (7). Additionally, it has been reported that antibiotics can inhibit members of the bee gut microbiota and cause bees to starve (14, 15). This has led to an increasing concern as well as interest in the investigation of alternative, effective, and novel control methods of AFB, including biological control agents with various modes of action, which could be used for apiculture.

Previous initiatives have identified various bacteria isolated from apiarian (16–19) and non-apiarian sources (7, 20, 21) that exhibited antagonistic activity against *P. larvae*, suggesting that bacteria from diverse environmental sources may have potential for controlling AFB in honeybees. However, most of these studies were limited to laboratory-based *in vitro* assays. For example, Bartel et al. (22) showed that several species of *Bacillus* (including *B. licheniformis*) can inhibit *P. larvae* in co-culture. A few studies have evaluated these inhibitory effects in honeybee larvae. Al-Ghamdi et al. (11) showed that the addition of *Lactobacillus kunkeii* and *B. licheniformis* to the larval diet significantly reduced the mortality of *P. larvae*-infected larvae (11). Despite these findings, a systematic investigation of bacteria for their biocontrol potential against *P. larvae* across multiple experimental levels: from initial *in vitro* antagonism screening to identify bacterial strains, followed by *in vivo* larval infection assays, and then evaluating the safety of the selected bacterial treatment in adult honeybees and their gut microbiota has not been explored. This important gap in the development and validation of treatments for AFB infections presents an opportunity to explore the use of biologicals in beekeeping.

Considering this, we investigated the biocontrol potential of hive-associated *Bacillus* species in enhancing the survival of honeybee larvae during *P. larvae* infection. First, we isolated bacteria from AFB-infected comb samples and taxonomically identified them. Secondly, we tested their ability to inhibit *P. larvae* strains *in vitro*. Thirdly, promising candidates from the *in vitro* assays were further examined for their ability to improve larval survival using laboratory-reared honeybee larvae. Finally, the best bacterial candidate was selected to assess its effect on the survival of adult bees and the gut microbiome, using caged adult worker honeybees in the laboratory.

## 2 Materials and methods

### 2.1 Source and isolation of bacterial strains

Three different *P. larvae* strains used in this study were acquired from two sources: *P. larvae* strains Y-3650 (NRRL B-3650) and 368 (ATCC-25368) from the University of Nevada, Las Vegas (23), and 25747 (ATCC-25747) from the American Type Culture Collection (ATCC). Among these strains, Y-3650 and 25747 belong to Enterobacterial Repetitive Intergenic Consensus (ERIC) group I, whereas 368 belongs to ERIC group IV (24). Another strain of *P. larvae* (ONT-R-1C (strain 55)) was isolated by our lab and is described in the Results section. A *Lactobacillus* strain used was received from NCIMB (NCIMB 15254, NCIMB Ltd, Wellheads Place, Dyce, Aberdeen, AB21 7GB). The *P. larvae* strains were retrieved from their glycerol stocks on 1mg/L of thiamine hydrochloride Brain Heart Infusion (BHI) agar as described in Phan et al. (25), and the *Lactobacillus* strain was streaked onto de Man, Rogosa and Sharpe (MRS) (Catalog#1.10660, GranuCult prime, EMD Millipore Corporation) media plates with 5% fructose. Bacteria for inhibition screening were isolated from two different sources: adult bee guts and AFB-infected comb samples, which included small pieces of honeycomb from 4 different frames from a hive infected with AFB, containing dark, perforated cell caps with debris- hereafter called “AFB-infected comb samples”. Under sterile conditions, these samples were ground in mortars and pestles in 1mL of sterile Phosphate buffer at pH 7 (PBS, Catalog#BP399-1, Fisher Scientific, USA). The ground mixtures were then serially diluted (10^0^ to 10^-4^) with PBS, and 100 µL of each dilution was plated onto MRS and BHI media, respectively. The BHI plates were incubated at 37°C for 72 hours, and the MRS plates were incubated in an anaerobic chamber at 37°C for 72 hours. The bacterial isolates were sub-cultured by the streak method, and the purified colonies were used to make liquid cultures in BHI or MRS broths.

DNA was extracted using the Quick-DNA HMW MagBead Kit (Catalog #D6060, Zymo Research), following the manufacturer’s protocol. The DNA samples were quantified using a Qubit™ Flex Fluorometer (Catalog#Q33327, Invitrogen Life Technologies, CA, USA), followed by 16S rRNA gene amplification using a standard protocol described by Amin et al. (26). The amplified PCR products were verified by gel electrophoresis, purified using the GeneJET PCR Purification kit (Catalog#FERK0701, ThermoFisher Scientific, USA), and were sequenced by Eurofins Genomics, DNA Sequencing Lab, Louisville, USA. The obtained DNA sequences were taxonomically assigned using Geneious Prime Software^®^ 2025.1.2 (https://www.geneious.com). For the *P. larvae* strain [ONT-R-1C (strain 55)], DNA was sent to Plasmidsaurus for whole-genome sequencing. The sequences of all these strains were submitted to GenBank.

### 2.2 *In vitro* assays for screening the inhibitory activity against *P. larvae* strains

Bacterial lawns of four *P. larvae* strains (Y-3650, 25747, 368, ONT-R-1C (strain 55)) were prepared following the protocol described by Phan et al. (25). The lab-isolated *Bacillus* strains were streaked from their respective glycerol stocks onto BHI plates and the *Apilactobacillus* strain and the acquired *Lactobacillus* strain onto MRS plates, then incubated as described above. Bacterial cultures were prepared by incubating *Bacillus* strains for 24 hours and *Lactobacillus* for 48 hours in a 37°C shaking incubator at 120 rpm. Both bacterial cultures and cell-free lysates were evaluated to screen inhibitory bacteria against *P. larvae* strains, using agar well diffusion (27–29) and agar spot assays (30). For the agar well diffusion assays, 100 µL of the bacterial cultures was inoculated into the wells. For the agar spot assays, the bacterial cultures were centrifuged at 100 rpm for 2 minutes, and the resulting supernatants were filtered through 0.22 µM membrane filters to obtain lysates; subsequently, 20 uL was spotted on the bacterial lawns. The plates were incubated at 37°C for 24 hours with the lids up. Inhibition of *P. larvae* by bacterial cultures or lysates was determined by measuring the inhibition zones formed on a bacterial lawn on a solid agar plate. Only a subset of the isolated strains were tested, with all isolates being tested on at least three *P. larvae* strains. *Bacillus licheniformis* (ONT-R2-ASx) was selected for further characterization because its lysate inhibited all *P. larvae* strains.

### 2.3 Characterization of *B. licheniformis* (ONT-R2-ASx) lysate antimicrobial activity using *in vitro* spot plate assays

Liquid culture of *B. licheniformis* (ONT-R2-ASx) was prepared as described above (see Method section). Then, a subculture was prepared by adding the overnight culture to BHI broth at a 1:10 ratio in a sterile flask and incubating for 24 hours. The bacterial mixture was then centrifuged at 1000 rpm for 3 minutes, after which the supernatant was filtered through a 0.45 μM membrane filter, followed by a 0.22 μM membrane to obtain the lysate. A portion of the 0.22 μM filtrate lysate was then passed through a 100 kDa molecular-weight cut-off (MWCO) membrane filter and likewise through 50 kDa and 30 kDa MWCO filters, respectively. Then, a total of four different filtrates/lysate sizes: (A) Less than 0.22 µM–greater than 100 kDa; (B) Less than 100 kDa–greater than 50 kDa; (C) Less than 50 kDa–greater than 30 kDa; (D) Less than 30 kDa and their 1:10 dilutions, were tested against the *P. larvae* strains Y-3650, 368, and strain 55 by spotting 20 µL onto their bacterial lawns. Each lysate size and its ten-fold (1:10) dilutions were tested in triplicate (n=3). The extent of bacterial inhibition was determined by measuring the diameter of the inhibition zone (cm) formed on the bacterial lawn on a solid agar plate. Statistical analyses were conducted in R (v4.5.2). A two-way ANOVA was performed to assess the effects of the *B. licheniformis* ONT-R2-ASx lysate against different *P. larvae* strains, lysate sizes, and their interaction on inhibition zones. To compare the inhibition zones of different lysate sizes within each *P. larvae* strain, means were calculated, and pairwise comparisons were performed using Tukey’s honestly significant difference (HSD) test with adjustment for multiple comparisons. Statistical significance was determined at *P* < 0.05.

### 2.4 Testing the effect of proteinase K, royal jelly, and temperature on the antimicrobial activity of *B. licheniformis* ONT-R2-ASx (50–30 kDa) lysate

The *B. licheniformis* ONT-R2-ASx (50–30 kDa) lysate was further used for stability tests to understand its composition. The susceptibility to proteolytic enzyme (proteinase K), royal jelly (Feed A, fed to the larvae during laboratory rearing), and heat treatment was evaluated. ONT-R2-ASx lysate was treated with proteinase K at a concentration of 100 μg/mL at 37°C for 30 mins, after which the treated lysate was tested for its antimicrobial activity against *P. larvae* strains (31, 32). To evaluate whether Feed A affects the lysate’s antimicrobial effectiveness the lysate and Feed A (1:1) were incubated at 37°C for 30 mins, and this mixture was spotted on the lawns of the *P. larvae* strains. To analyze thermal stability, the lysate was serially diluted (10^0^, 10^-1^, 10^-2^, and 10^-3^), and each dilution was heat-treated at 100°C for 10 mins. The heat-treated tubes were kept in ice immediately before spotting them onto the bacterial lawns. Then, 20 μL of each of the above-mentioned treatments was spotted on the lawns of *P. larvae* strains as mentioned above. After incubation for 24 hours, the diameter of the inhibition zone formed on the bacterial lawn was measured. Each treatment was screened independently in triplicate plates (n=3). Inhibition zone data were analyzed using two-way ANOVA, and mean inhibitions within each *P. larvae* strain were compared using Tukey’s honestly significant difference (HSD) test, using R software (v4.5.2). Statistical significance was determined at *P* < 0.05.

### 2.5 *In vivo* larval infection assays using laboratory-reared honeybee larvae

Larval infection assays were conducted using three bacterial strains that were selected based on how well they inhibited *P. larvae in vitro*. *B. zhangzhouensis* (strain ONT-R-2D), hereafter called “2D *Bacillus*”, was selected since its culture inhibited *P. larvae* strains Y-3650 and 368. The acquired NCIMB *Lactobacillus* strain, hereafter called “NCIMB Lacto”, inhibited all tested *P. larvae* strains *in vitro*. *B. licheniformis* (ONT-R2-ASx) was selected because its lysate inhibited two *P. larvae* strains, Y-3650 and strain 55. Among the *P. larvae* strains, Y-3650 was selected for the study since it belongs to ERIC-group I (24) and is reported to be one of the ERIC groups driving the global AFB outbreak situation (33). *P. larvae* strain 55 was also selected since it was isolated from the AFB-infected comb samples from our region. Each infection assay included four treatment groups: no bacteria, pathogen alone, pathogen and inhibitory bacterium or lysate, and inhibitory bacterium or lysate alone.

First-instar larvae were reared following standard procedures (34, 35). Larvae were fed a mixture of 10 µL pre-warmed artificial larval diet “Feed A” (36) and 1 µL of treatment-solution (e.g., bacterial inoculum). The solution contained either BHI, 100 CFU/µL each bacterium in BHI, bacterial culture lysate, or size-selected lysate. The temperature and humidity of the larva-rearing incubator were maintained at 35°C and 95–100%, respectively. 12 larvae were used for each of the four different treatments, and each treatment was repeated three times (n=36 larvae). Mortality and abnormal symptoms were visually observed and recorded every day. The larvae were considered dead when the larval color became dark or they had no motion, and were removed daily from the plates. The difference in the survival of the tested larvae throughout the experiments was analyzed by using log-rank tests with all pairwise comparisons with Benjamini-Hochberg multiple test correction. All the statistical analysis were performed and compared in R (v4.5.2) using the “survival” and “survminer” packages. Statistical significance was determined at *P* < 0.05.

### 2.6 Testing the effect of *B. licheniformis* ASx lysate in adult worker honeybees reared in laboratory conditions

Cage experiments were performed with modifications to standard cage-rearing methods (37, 38). Sixteen random healthy brood frames containing capped brood were collected from three different hives/colonies from our research apiary located at the University of Idaho (Moscow, Idaho, USA) and transferred to the laboratory in nucleus colony (Nuc) boxes. The queens used in these three hives were received from Northern California, USA. Frames were incubated at 33°C and 60% relative humidity for 24 hours until adult bees emerged. On day 0 (D0), the newly emerged adult (NEWs) worker bees were caged in custom-printed plastic insect cages sized 20 x 14.5 x 14.5 cm: 100 bees per cage. They were maintained at 33°C and 60% RH, and different treatments were provided on D0 via inverted 50 mL conical tubes with the open end covered with sterile fabric. Comb collected from 16 frames were mixed and provided in all cages to provide a source of hive microbes. In total, 13 cages was used in the experiment: 4 treatments with 3 replicate cages per treatment, and one cage was used for measuring feed evaporation rates. On days D0, D6, and D12, bees were fed 10 mL either 40% sugar syrup only (control); 40% sugar syrup mixed with laboratory-grade tetracycline (Catalog T252525G, TCI America ™, Fisher Scientific, USA) at the doses of 0.1 mg/mL; 40% sugar syrup mixed with commercial Terra-Pro mediated Terramycin treatment (MLL: 25-055; Item#DC-550; Mann Lake Ltd. Hackensack MN) at the doses of 0.014 gram/mL; or ASx lysate: 40% sugar syrup at the ratio of 1:10. All cages received ad libitum control 40% sugar syrup between treatments, and their consumption was recorded at 24-hour intervals throughout the experiment. Survival was calculated daily based on the remaining bees after sampling. The survival differences among bees across treatments were analyzed using log-rank tests, with all pairwise comparisons corrected for multiple testing using the Benjamini-Hochberg method. All statistical analysis were performed and compared in R using the “survival” and “survminer” packages, and statistical significance was determined at *P* < 0.05.

The gut microbiome was assessed for sterile NEWs before treatment, the comb placed in cages, and all treatment groups on days D5, D11, and D17. Additionally, we evaluated the impact of laboratory cage rearing by including a mark-recapture treatment group. Around 200 NEWs from the healthy brood frame were marked on D0. A tiny dot of non-toxic, water-based acrylic paint was applied to the center of the thorax of each of these 200 NEWs, and the paint was allowed to dry completely for around 30 min before introducing them to a hive in our apiary. The marked bees were later collected (n=3 pools of 6 bees) to extract guts. On days D5, D11, and D17, pools of 15 NEWs were randomly collected from all 12 cages. Digestive tracts were dissected on PBS and stored at -80°C until DNA extraction was performed. DNA was extracted using ZymoBIOMICS DNA Microprep kits (Catalog # D4301, Zymo Research) according to the manufacturer’s protocol.

DNA was sequenced by Plasmidsaurus using Oxford Nanopore (ONT) R10.4.1 flow cells with long-read sequencing libraries constructed using v14 library prep chemistry and in-house 16S gene primers. Taxonomic identification was completed by Plasmidsaurus using emu (v3.5.1) against rrnDB (v5.6) and NCBI targeted Loci databases (https://www.plasmidsaurus.com/). Reads were submitted to GenBank (PRJNA1377638). All statistical analysis were conducted in R (v4.5.2) using custom scripts. Taxonomic data were imported and analyzed using phyloseq (v1.54.0). Beta diversity was assessed via principal coordinates analysis (PCoA) with Bray-Curtis dissimilarity matrices, and community similarity was calculated using Jaccard distances with the vegan package (v2.7.2). Differences in community composition among groups were tested by permutational multivariate analysis of variance (PERMANOVA; adonis2, vegan) on Bray-Curtis dissimilarities with 999 permutations, both across all sample types and, separately, among the caged treatment groups. Differential abundance analyses were performed with default parameters in DESeq2 (v1.50.2) and ANCOM-BC2 (v2.12.0), with control as the reference group (39).

## 3 Results

### 3.1 Bacterial strains isolated from bees and AFB-infected comb inhibit *P. larvae* growth

We isolated and characterized ten bacterial strains from honeybee guts and AFB-infected comb samples by culturing and sequencing isolates. Among them, two (one *Bacillus* strain and one *Apilactobacillus* strain) were extracted from bee guts collected from bees from our apiary in Moscow, Idaho, and eight *Bacillus* strains, including *Bacillus zhangzhouensis*, *B. subtilis*, *B. pumilus*, *B. amyloliquefaciens*, *B. licheniformis*, and *B. mojavensis*, from AFB-infected comb. In addition, a *P. larvae* ONT-R-1C (strain 55) was isolated from the AFB-infected comb sample, which was characterized by whole-genome sequencing. The sequences of these isolates were deposited at GenBank under Bioproject PRJNA1377638, and their accession IDs are provided in **Supplementary File S1**. P*. larvae* strain 55 was identified as Sequence Type (ST)-15 in the *P. larvae* MLST database (40), belonging to ERIC group-I (41).

The isolated *Bacillus* and *Apilactobacillus* strains, and a *Lactobacillus* strain from NCIMB GenBank, were tested for their ability to inhibit four different *P. larvae* strains: Y-3650, 25747, ONT-R-1C (strain 55), and 368. Among the bacterial isolate–*P. larvae* strain combinations tested, 5/10 strains of the bacteria we isolated, and the *Lactobacillus* strain from NCIMB GenBank, strongly inhibited one or more *P. larvae* strains when co-cultured on agar plates; 8/11 showed overgrowth on the lawns of one or more *P. larvae* strains, and 1/11 had no effect at all (**Figure 1A**; **Supplementary Figure S1**). Overgrowth was characterized by the expansion of the “inhibitory” bacteria from the inoculation spot. When we compared inhibition from cell cultures to inhibition from spent media (cell-free lysates), we found that *P. larvae* was often inhibited by both the culture and the cell-free lysate from the same culture (**Figure 1**). We also found that these isolates have different inhibitory effects on different *P. larvae* strains. For example, the culture of *B. zhangzhouensis* (ONT-R-2D) inhibited *P. larvae* Y-3650 and 368 but had overgrowth on 25747 and ONT-R-1C (strain 55). Both *B. pumilus* isolates that we tested had overgrowth on *P. larvae* lawns. NCIMB *Lactobacillus* strain and *B. licheniformis* had inhibitory effects on the tested *P. larvae* strains, either with their cultures (cells), with lysates (without cells), or with both cultures and lysates. Among all the tested bacterial strains, *B. licheniformis* (ONT-R2-ASx) was among the strongest inhibitors of *P. larvae*, but only the lysate inhibited *P. larvae* Y-3650 and strain 55, whereas the cultures had overgrowth on those lawns. Both its lysate and culture inhibited *P. larvae* 368 (**Figure 1B**). Further characterization of *B. licheniformis* (ONT-R2-ASx) lysate was conducted to evaluate its inhibitory properties.

**Figure 1:**
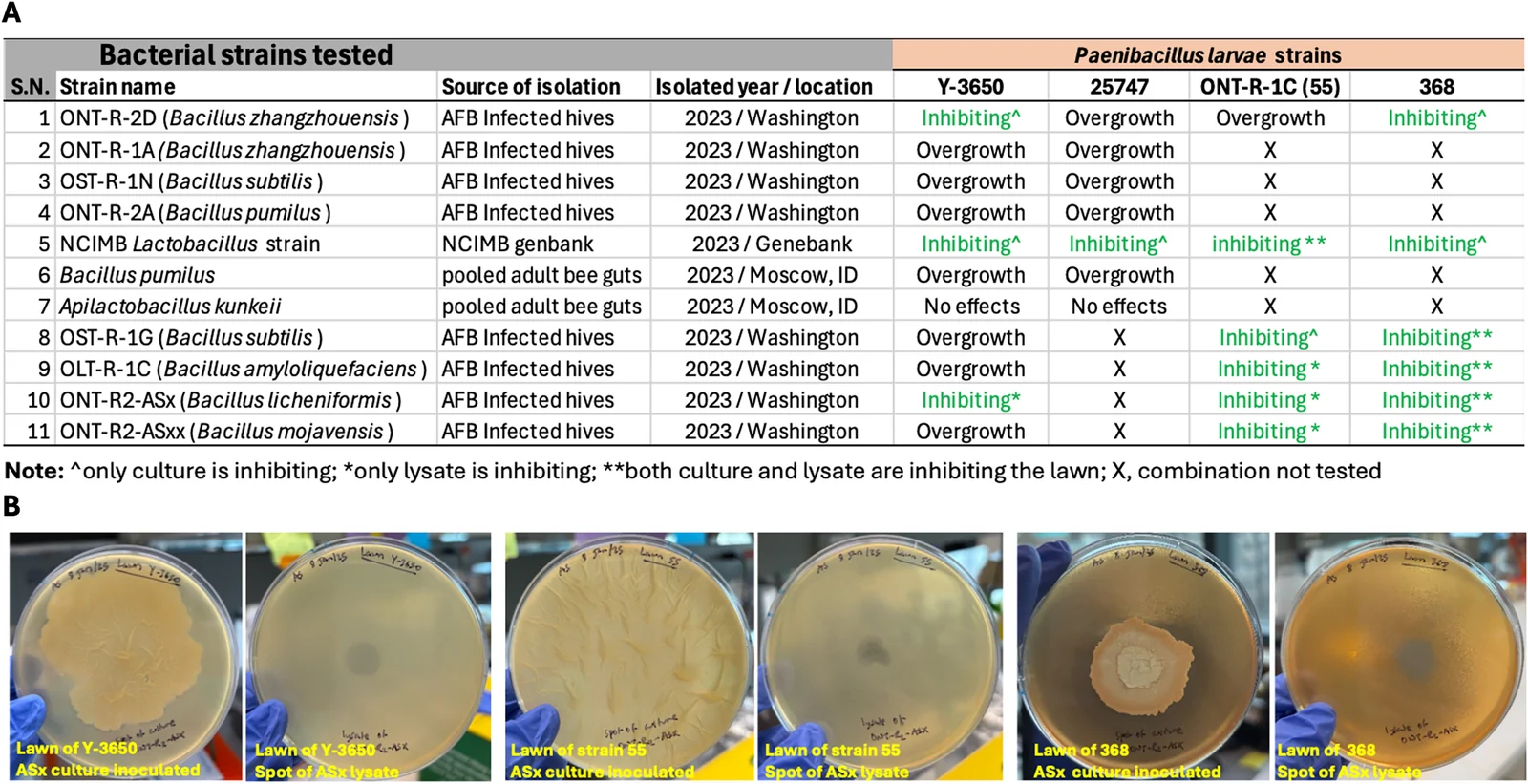
Bacterial isolates that inhibit *P. larvae* strains *in vitro*: **(A)** a list of bacterial strains used in the study, their sources and year of isolation, locations from where the samples were received, and their effects on *P. larvae* strains. **(B)** Example showing the effect of cultures and lysates of the strain *B. licheniformis* (ONT-R2-ASx) against the *P. larvae* strains. An “X” indicates that the combination was not tested.

### 3.2 Characterization of *B. licheniformis* (ONT-R2-ASx) lysate

The cell-free lysate of *B. licheniformis* (ONT-R2-ASx) was investigated by testing fractions separated by size: 0.22 μM–100 kDa, 100–50 kDa, 30–50 kDa, and less than 30 kDa. These fractions were analyzed for their inhibitory effect on the *P. larvae* strains by spot plating. The spot plate assays showed that all the non-diluted lysate fractions except those containing proteins less than 30 kDa in size inhibited the growth of *P. larvae* (**Figure 2**; **Supplementary Figure S2**; **Table 1**). We noticed significant reductions (*P* < 0.05) in the size of the zone of inhibition in all the *P. larvae* lawns as filter size decreased (**Table 1**). The ten-fold dilutions of each fraction were also tested to determine the potency of the antimicrobial compounds present; two lysate fractions (0.22 μM–100 kDa) and (100–50 kDa) showed inhibition only on the lawn of *P. larvae* strain 55, and the inhibitory activity was lost in the 50–30 kDa fractions (**Table 1**; **Supplementary Figure S2**).

**Figure 2:**
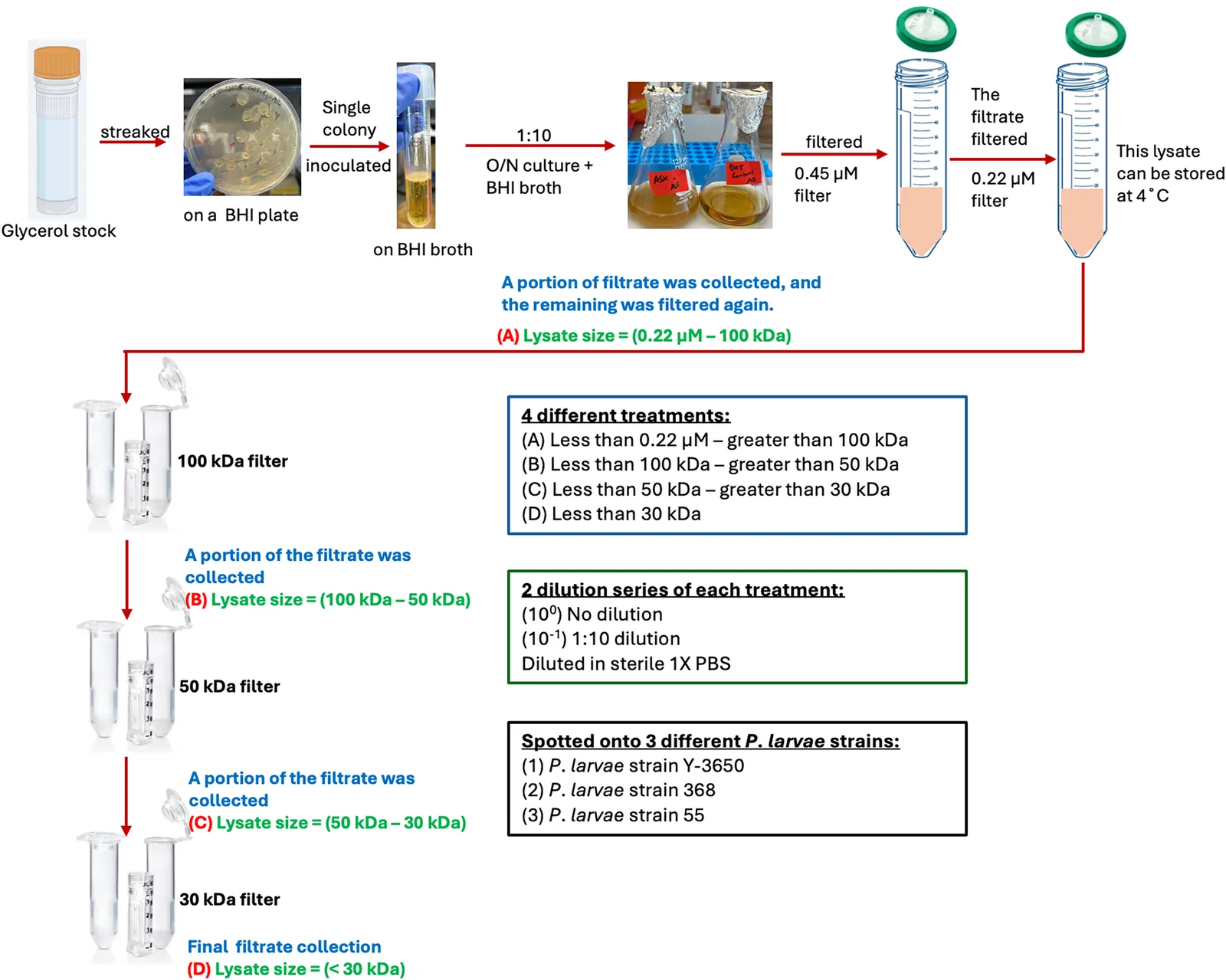
Testing different *B. licheniformis* (ONT-R2-ASx) lysate fractions on the lawn of three different *P. larvae* strains. The flow diagram shows the steps from streaking the *B. licheniformis* (ONT-R2-ASx) strain from its glycerol stock and collecting different sizes of filtrate lysates from its culture, which were then spotted onto the lawns of three *P. larvae* strains.

**Table 1:** Inhibition of different *P. larvae* strains by *B. licheniformis* (ONT-R2-ASx) lysate fractions of different molecular weight ranges and dilutions/concentrations.

*P. larvae* strains	Dilution of lysate	Inhibition zone diameter (in cm) (Mean ± SEM) (n=3)			
		0.22uM–100kDa	100–50kDa	50–30kDa	Less than 30kDa
Y-3650	10^0^	1.57 ± 0.03 d	1.37 ± 0.08 c	1.07 ± 0.03 b	0.00 ± 0.00 a
	10^-1^	0.00 ± 0.00 a	0.00 ± 0.00 a	0.00 ± 0.00 a	0.00 ± 0.00 a
368	10^0^	1.50 ± 0.05 d	1.15 ± 0.00 c	0.63 ± 0.03 b	0.00 ± 0.00 a
	10^-1^	0.00 ± 0.00 a	0.00 ± 0.00 a	0.00 ± 0.00 a	0.00 ± 0.00 a
Strain 55	10^0^	2.05 ± 0.07 e	1.85 ± 0.02 d	1.55 ± 0.02 c	0.00 ± 0.00 a
	10^-1^	0.59 ± 0.00 b	0.59 ± 0.01 b	0.00 ± 0.00 a	0.00 ± 0.00 a

Values represent mean inhibition zone diameters in centimeters (cm) ± Standard Error of the Mean from three independent replicates.

Within each strain, values sharing the same letter are not significantly different (*P* < 0.05).

### 3.3 Influence of proteinase K enzyme, royal jelly, and temperature on the antimicrobial activity of ASx 50–30 kDa lysate

To further investigate the components of the ASx 50–30 kDa lysate causing antimicrobial activity against *P. larvae*, we exposed the lysate fraction to various treatments and measured any losses in antimicrobial activity. Across all *P. larvae* strains, the antimicrobial activity (inhibition) was completely lost when the ASx 50–30 kDa lysate was exposed to proteinase K at a concentration of 100 μg/mL and was significantly different from all other treatments (*P* < 0.05) (**Table 2**; **Figure 3**). After heat treatment, the antimicrobial activity was significantly reduced for *P. larvae* strain 368 but had no significant effect in Y-3650 and strain 55, when compared to their respective controls (**Table 2**; **Supplementary Figure S3**). The royal-jelly treated lysates did not differ significantly from the controls in all these strains (**Table 2**; **Figure 3**).

**Table 2:** Testing the ability of different treatments of ASx 50–30 kDa lysates to suppress *P. larvae* strains. Values are reported as Mean ± SEM (cm).

Treatment	Inhibition zone diameter (in cm) (Mean ± SEM) (n=3)		
	Y-3650	368	Strain 55
ASx 50–30 kDa Control	1.10 ± 0.00 a	0.63 ± 0.03 a	1.43 ± 0.03 a
ASx 50–30 kDa PK-treated	0.00 ± 0.00 b	0.00 ± 0.00 b	0.00 ± 0.00 b
ASx 50–30 kDa RJ-treated	1.09 ± 0.01 a	0.55 ± 0.03 ac	1.40 ± 0.06 a
ASx 50–30 kDa Heat-treated	1.09 ± 0.00 a	0.44 ± 0.03 c	1.43 ± 0.03 a

Different letters indicate significant differences (*P* < 0.05) among treatments within each strain. Treatments sharing the same letter are not significantly different (*P* > 0.05). Treatments with different letters are significantly different (*P* < 0.05). PK-treated = proteinase K-treated; RJ-treated = royal jelly-treated

**Figure 3:**
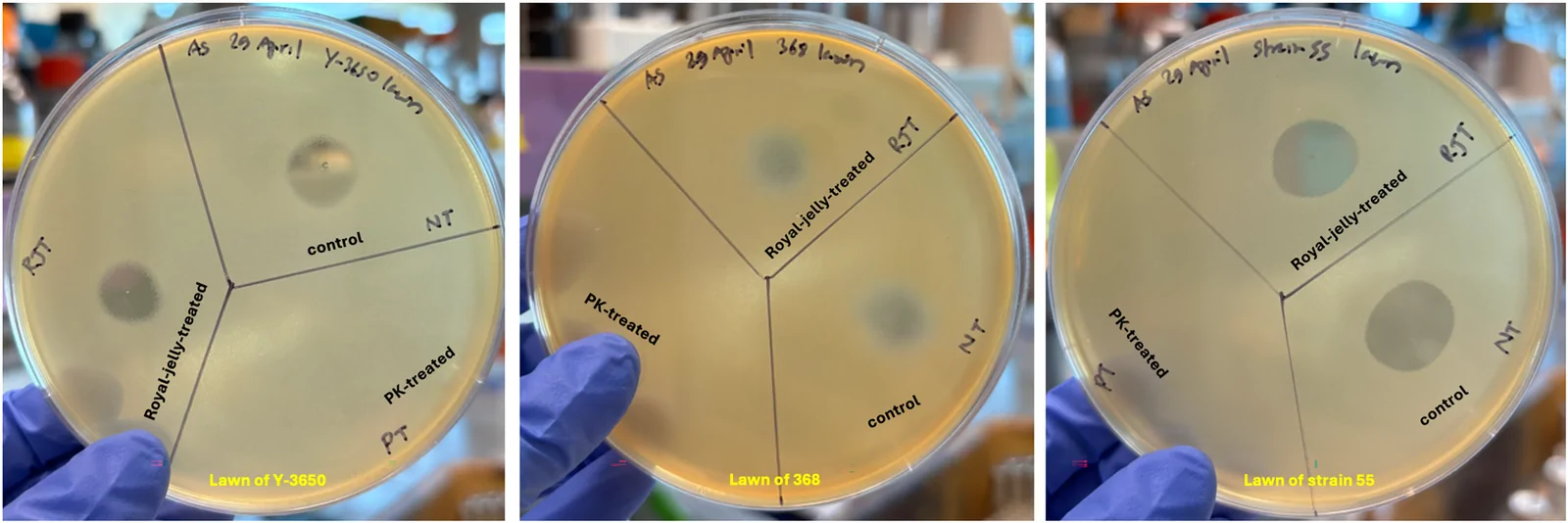
Representative plates showing different treatments of *B. licheniformis* (ONT-R2-ASx) 50–30 kDa lysate on the lawns of *P. larvae* strains. Pictures of heat-treated spot assays are shown in **Supplementary Figure S3**. PK-treated = proteinase K-treated.

### 3.4. The effects of 2D *Bacillus* and NCIMB Lacto cultures on the laboratory-reared honeybee larvae

The cultures of both strains—the NCIMB Lacto and 2D *Bacillus* showed inhibitory effects in the *in vitro* assays. Therefore, these strains were tested in honeybee larvae to evaluate their effectiveness against the *P. larvae* Y-3650 *in vivo*. The larval experiments using 2D *Bacillus* and Y-3650 showed no significant differences in larval survival when larvae were fed only 2D *Bacillus* cultures (58%) and both *P. larvae* Y-3650 and 2D *Bacillus* together (42%), compared with those fed only BHI broth (72%) [*P*=0.15] and [*P*=0.05], respectively. However, they showed significant differences in survival when fed these bacteria when compared with the larvae inoculated only with *P. larvae* Y-3650 (6%) [*P*=0.0045] and [*P*=0.021], respectively (**Figures 4A–E**).

**Figure 4:**
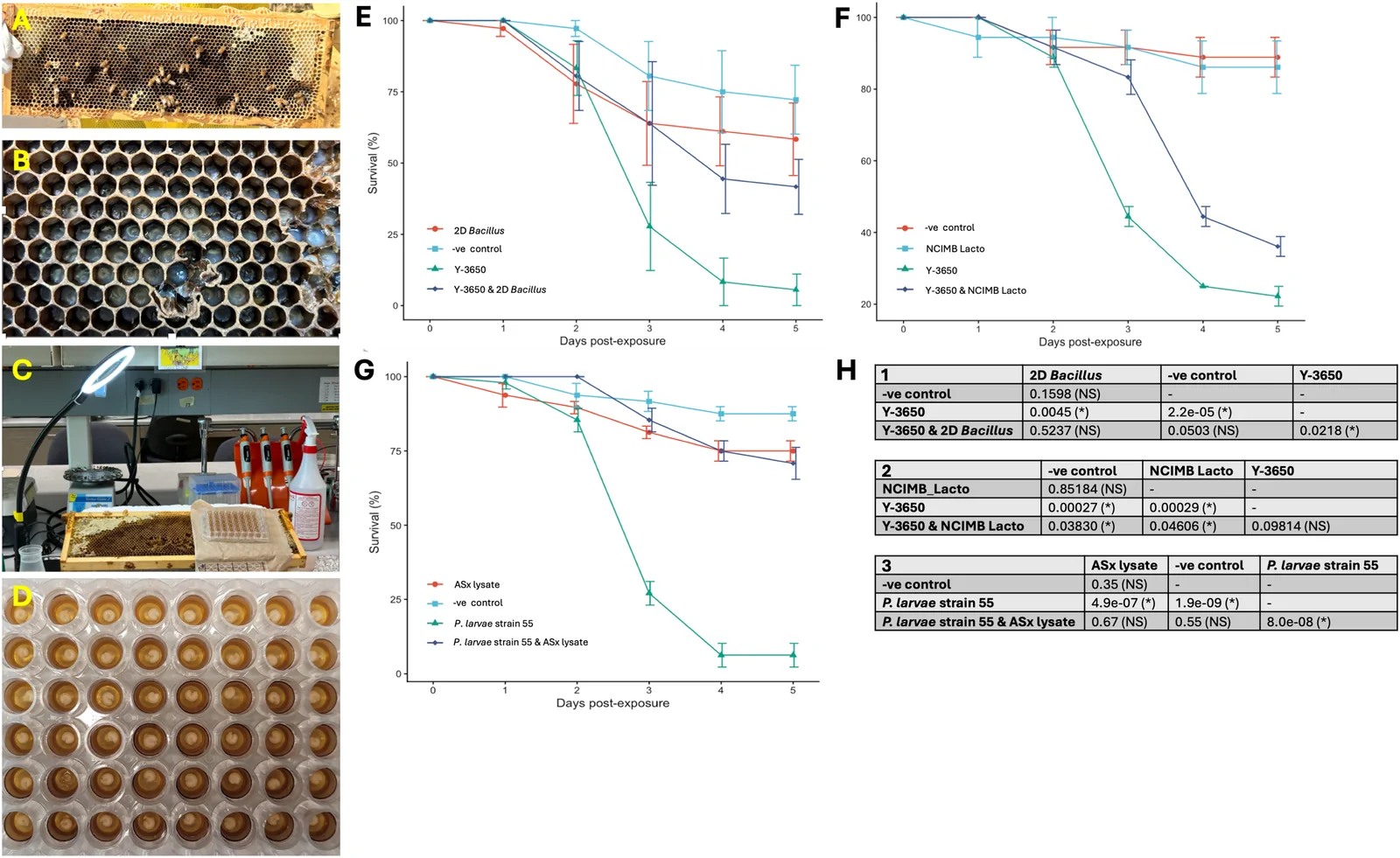
Testing the potential of different antimicrobial strains or *B. licheniformis* ONT-R2-ASx lysate (ASx lysate) to protect larvae against two *P. larvae* strains. **(A)** A-frame collected from our apiary in Moscow, Idaho; **(B)** Frame with first-instar larvae; **(C)** Lab set-up for larval grafting; **(D)** Larvae in a 48-well plate after 24 hours of grafting; **(E)** 2D *Bacillus*, *P. larvae* Y-3650, and their combinations; **(F)** NCIMB Lacto, *P. larvae* Y-3650 and their combination; and **(G)** Results showing the ASx lysate could improve the survival of honeybee larvae during the infection by *P. larvae* strain 55. Standard error bars are shown; **(H)** Results of the statistical analysis of the three infection assays, mentioned above, analyzed in R using the “survival” and “survminer” packages where the statistical significance was determined at *P* < 0.05 (1) 2D *Bacillus*, Y-3650, and their combination; (2) NCIMB Lacto, Y-3650, and their combination; and (3) ASx lysate, *P. larvae* strain 55, and their combination.

Inoculation with NCIMB Lacto alone showed no significant difference in larval survival from the BHI controls [*P*=0.85]. Larvae that were co-inoculated with both NCIMB Lacto and *P. larvae* Y-3650 had significantly lower survival than those fed NCIMB Lacto alone [*P*=0.04], and no significant difference from the larvae fed only with *P. larvae* Y-3650 [*P*=0.09] (**Figures 4A–D, F, H.2**).

### 3.5 The antimicrobial peptide of ASx lysate has biocontrol potential in improving the survival of honeybee larvae during *P. larvae* infection

As reported earlier, ASx lysate has an antimicrobial compound that can inhibit *P. larvae* strains in *in vitro* assays. To determine if these antimicrobial effects are relevant to honeybees, infection assays were conducted on laboratory-reared honeybee larvae. Under standard larval rearing procedures, we found good survival of grafted bees [88% (42/48)] up to 5 days post-grafting. Larvae infected with approximately 100 cells of *P. larvae* strain 55 had poor survival, with only 6.25% (3/48) remaining at the end of the experiment. This was significantly different than larvae treated with ASx lysate [*P*= 4.9e-07, log-rank test with BH correction, n=1152, df=3] or infected with *P. larvae* strain 55 and treated with the ASx lysate [*P*=8.0e-08, log-rank test with BH correction, n=1152, df=3]. When compared with the control-treated larvae, we found no significant difference in the survival of larvae infected with *P. larvae* strain 55 and treated with the ASx lysate [70.8% (34/48)] [*P*=0.55] or when only treated with the ASx lysate alone [75% (36/48)] [*P*=0.35] (**Figures 4A–D, G, H.3**).

### 3.6 Mortality of adult honeybees treated with antibacterial compounds

The *in vivo* larval infection assay showed that ASx lysate protected honeybee larvae during *P. larvae* infection. To evaluate the safety of treating honeybees with ASx lysate, cage experiments were conducted, where bees were fed with either sugar syrup or syrup containing either tetracycline, Terra-Pro, or the ASx lysate (**Figure 5A**). On Day 5 post-first inoculation, the survival percentage of the adult bees treated with ASx lysate (94.4%) and Terra-Pro (95%) was significantly different from that of the non-treated negative control bees (97.4%; *P*=0.016, log-rank test with BH correction, n=6600, df=3; where “n” represents the total number of time-point records generated from repeated observations of the individual bees), whereas the survivability percentage of tetracycline-treated bees (97.6%) was similar to the controls (*P*=0.192, log-rank test with BH correction, n=6600, df=3) (**Figure 5B**). On Day 5, 15 bees were pooled from each cage for microbiome analysis, and on Day 6, the second dose of the treatments was fed to the remaining bees. On Day 11 post-second inoculation, similar results were obtained as on Day 5; there was no significant difference in the survival percentage of adult bees treated with ASx lysate (67.6%) versus Terra-Pro (72.3%) [*P*=0.965, log-rank test with BH correction, n=13200, df=3]; however, both treatments had significantly different survival percentages when compared with controls and tetracycline-treated bees (91.8% and 85.1% respectively) [*P*=1.1E-14, 6.6E-13 respectively] (**Figure 5C**). Again, 15 bees were pooled per cage on Day 11, and the third dose of the treatments was fed to the leftover bees. On Day 17 post-third inoculation, the results were different. The survival percentages of ASx lysate-treated, Terra-Pro-treated, and tetracycline-treated bees were significantly different than the survival percentages of the non-treated control bees. Also, the survival percentages of ASx lysate- and Terra-Pro-treated bees were significantly different than that of tetracycline-treated bees (**Figure 5D**). The number of surviving bees at each sampling point is presented in **Supplementary File S2**.

**Figure 5:**
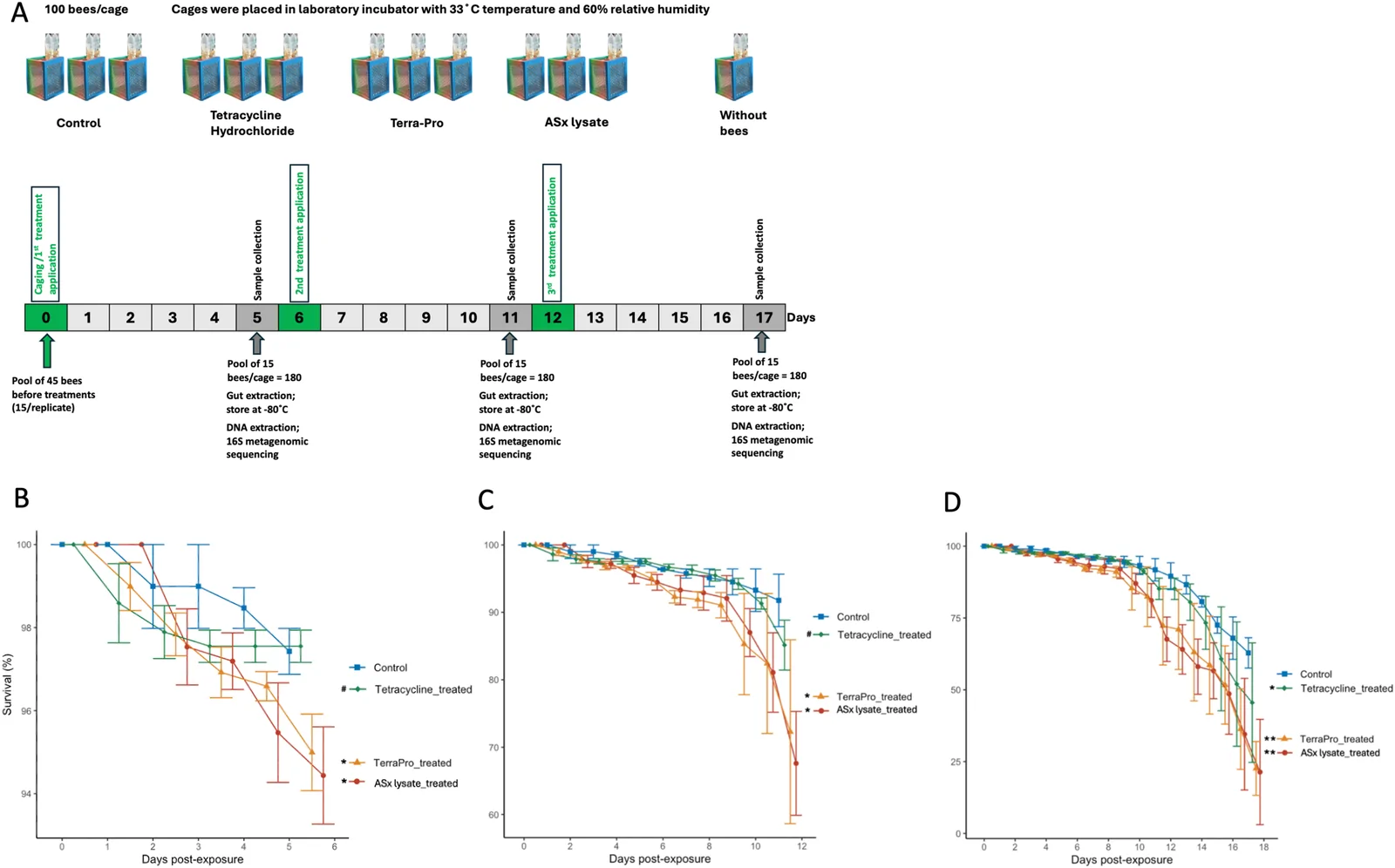
The potential impact of the ASx lysate in adult honeybees. **(A)** Study design and methodology used for testing the effect of ASx lysate, including lab-grade tetracycline and commercially available Terra-Pro, on newly emerged worker honeybees in cages under lab conditions. The study was conducted in September–October 2025. **(B–D)** Mean percentage survival of newly emerged adult worker bees (n=300/treatment) in the laboratory cages, fed with a total of 3X treatments of either tetracycline or TerraPro, or ASx lysate; **(B)** Bees caged received 1X treatments, from 0 to 5 days; **(C)** received 2X treatments from 0 to 11 days; **(D)** received 3X treatments from 0 to 17 days. # indicates the survival percentage is not significantly different from the control (*P* > 0.05); * indicates the survival percentage is significantly different from the control, and ** indicates the survival percentage is significantly different from both the control and tetracycline-treated bees (*P <*0.05).

### 3.7 The impact of treatments on the adult honeybee gut microbiome

We sequenced a total of 44 pools of dissected bee guts from days 0, 5, 11, and 17 using full-length 16S rRNA gene sequencing (**Figure 6**). The mean number of post-quality filtering reads was 4,824 ± 625 per sample with a mean quality score of 20.79 and a mean read length of 1,444 base pairs. Taxonomic classification identified 55 unique taxa comprising ≥ 0.1% relative abundance across all samples (**Supplementary File S3**: taxonomy_all_samples; **Supplementary File S4**: sequencing_stats_all). The overall taxonomic composition reflected the characteristic core microbiome of adult honeybee workers, with *Lactobacillus* and *Gilliamella* as the dominant genera. *Lactobacillus* was the most abundant genus in 38 of 44 samples, represented by 8 distinct species. In the six samples where *Lactobacillus* was not the dominant genus, *Gilliamella* (n=2), *Enterobacter* (n=2), *Serratia* (n=1), and *Tatumella* (n=1) were the most abundant instead. These genera were distributed across treatment groups, controls, and timepoints (NEWs, Comb, Day 5 Control, Day 5 Terra-Pro, and Day 17 ASx lysate) (**Supplementary File S4**: dominant_genus). However, *Lactobacillus* remained present as the second-ranked genus in these samples. *Bifidobacterium*, a core member of the honeybee gut microbiome, was not identified in any of the samples, including hive-reared and comb (**Supplementary File S4**: core_bacteria_presence).

**Figure 6:**
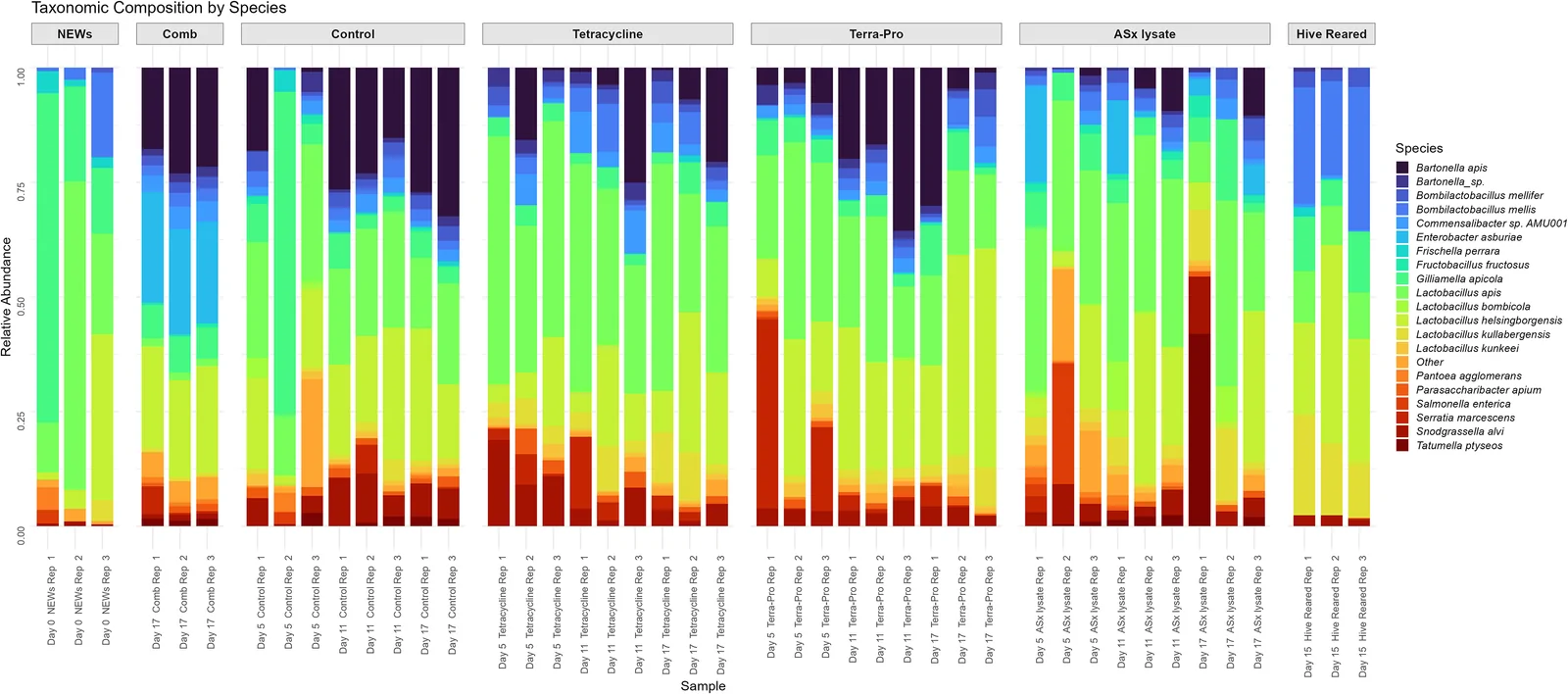
Taxonomic composition by species: Relative abundance of the top 20 most abundant bacterial species across all 44 samples, grouped by treatment and sampling day. Each bar represents an individual sample, with colors indicating different bacterial species. Sample types are organized left to right: NEWs (Newly Emerged Workers, collected on Day 0 for baseline estimate), comb (bacterial source material, collected on Day 17 for comparison), control (caged, untreated), tetracycline (caged, antibiotic treatment), Terra-Pro (caged, antibiotic treatment), ASx lysate (caged, lysate treatment), Hive-Reared (NEWs marked on Day 0 and returned to hive, collected on Day 15 comparison). Species of *Lactobacillus* comprised the majority of the community (mean ~52% relative abundance) with *Gilliamella* as the second most abundant genus. Control replicate 2, Day 17, is missing from the dataset due to the loss of bees for Day 17 sampling. *Lactobacillus mellis* and *L. mellifer* are displayed as *Bombilactobacillus mellis* and *B. mellifer* following updated classification for Firm-4 phylotype (42).

Principal coordinates analysis (PCoA) of Bray-Curtis dissimilarity captured 50.6% of the variation in the first two axes (PC1: 28.9%; PC2: 21.6%; **Figure 7**). Newly emerged workers, comb, and hive-reared bees each separated from the caged treatment groups (control, tetracycline, Terra-Pro, and ASx lysate), which overlapped within the central region of the ordination. These patterns were supported statistically: community composition differed significantly across sample types (PERMANOVA, R^2^ = 0.43, F=4.69, p=0.001; **Supplementary File S4**: permanova), driven primarily by the distinct newly emerged, comb, and hive-reared groups. Among the caged treatments alone, treatment nonetheless explained a significant fraction of the variation (R^2^ = 0.22, F=2.86, p=0.001; **Supplementary File S4**: permanova).

**Figure 7:**
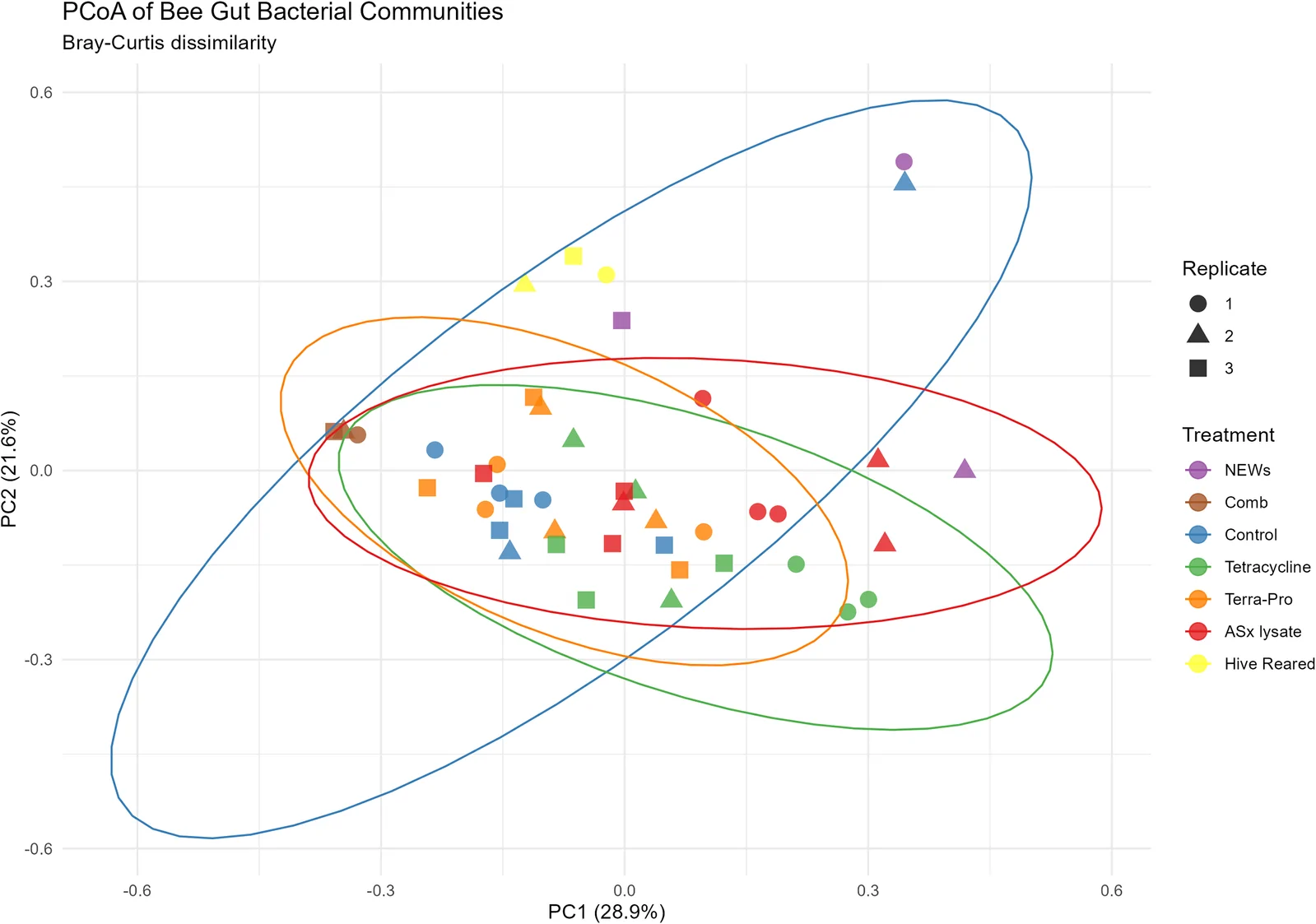
Principal coordinates analysis (PCoA) ordination of bacterial community composition using Bray-Curtis dissimilarity. Each point represents an individual sample, colored by treatment group and shaped by replicate number. 95% confidence ellipses are shown for treatment groups where they could be estimated; groups with insufficient replication are shown as points only. The first two axes capture 28.9% (PC1) and 21.6% (PC2) of the variation. NEWs, comb, and hive-reared bees separate from the caged treatment groups (control, tetracycline, Terra-Pro, and ASx lysate), which overlap centrally. Community composition differed significantly across sample types (PERMANOVA, R^2^ = 0.43, p=0.001) and among the caged treatments (R^2^ = 0.22, p=0.001), respectively. Antibiotic treatments: tetracycline and Terra-Pro cluster together. ASx lysate-treated samples show intermediate positioning between antibiotic-treated and untreated control samples. NEWs are distinct from all other groups. Hive-reared bees form a separate cluster from all treatments.

Jaccard distance analysis showed that the two antibiotic treatments were most similar to each other (0.28), while ASx lysate remained similar to controls (0.38). The NEWs were most distinct from all groups (0.67–0.74), and hive-reared bees differed from caged bees (0.45–0.71) (**Table 3**).

**Table 3:** Jaccard distances quantifying dissimilarities in bacterial community composition between sample types.

Sample type	NEWs	Control	Tetracycline	Terra-Pro	ASx lysate	Hive-reared	Comb
NEWs	0	–	–	–	–	–	–
Control	0.667	0	–	–	–	–	–
Tetracycline	0.688	0.485	0	–	–	–	–
Terra-Pro	0.676	0.394	0.28	0	–	–	–
ASx lysate	0.723	0.381	0.55	0.512	0	–	–
Hive-reared	0.68	0.633	0.45	0.522	0.711	0	–
Comb	0.743	0.514	0.6	0.548	0.537	0.565	0

Values range from 0 (identical communities) to 1 (different communities). Antibiotic treatments, tetracycline and Terra-Pro, show high similarity to each other. ASx lysate maintains greater similarity to untreated controls than to antibiotic treatments. The NEWs differ substantially from all developed microbiomes. Hive-reared bees show moderate distances from all caged treatments.

DESeq2 and ANCOM-BC2 were used to identify treatment-dependent differences in bacterial taxon abundance relative to untreated controls. By DESeq2, tetracycline was associated with seven differentially abundant taxa, Terra-Pro with 3, and ASx lysate with none, for a total of 10 associations across seven unique taxa (**Supplementary File S4**: deseq2_sig_taxa). ANCOM-BC2, a more conservative compositional method, identified no significantly different taxa at q<0.05, with one borderline taxon (*Lactobacillus kullabergensis*, q = 0.054) in the tetracycline versus control comparison (**Supplementary File S4**: deseq2_v_ ancombc2). Among the DESeq2-identified taxa, tetracycline also impacted the abundance of honeybee gut-specific *Lactobacillus kullabergensis* (**Supplementary File S4**: deseq2_v_ancombc2).

An analysis of the comb samples placed in all the cages revealed that 50–61% of the control and treatment bee gut taxa were also present in the comb (**Supplementary File S4**: comb_seeding). Hive-reared bees shared 90.9% of their taxa (10 of 11) with the same comb material used in caging experiments, despite exposure to natural hive comb (**Supplementary File S4**: comb_seeding).

## 4 Discussion

This study demonstrated that hive-associated *Bacillus* species have biocontrol potential against *P. larvae*, and that selected strains translate their antagonistic activity *in vitro* into improved larval survival following pathogen challenge. The selected *B. licheniformis* strain produced heat-stable antimicrobial activity associated with a nominal 30–50 kDa fraction, further supporting the involvement of proteinaceous antimicrobial component(s), although its efficacy varied among *P. larvae* strains. The minimal effect of the selected most promising *Bacillus* treatment on adult gut microbiome composition provides evidence that the treatment did not substantially disturb the adult gut microbiome. These findings support the potential of hive-associated *Bacillus* species as biologically based tools for AFB management.

The *Bacillus* species are not core members of the honeybee gut microbiome; however, they are commonly found in the beehive, honey, pollen, and hive materials (10). They are reported to be detected in small numbers in bee guts (11). Previous studies reported that *Bacillus* strains isolated from different apiarian sources such as honey, honeybee guts, and larvae exhibit antagonistic effects against various *P. larvae* strains *in vitro*. For example, *B. megaterium*, *B. licheniformis*, *B. subtilis*, and *B. cereus*, isolated from honey and honeybees, inhibited *P. larvae in vitro* (11, 16–19, 43). *B. licheniformis* showed inhibition against *P. larvae* genotypes ERIC I and IV, and *B. subtilis* inhibited *P. larvae* genotypes ERIC I, II, and IV (22). Likewise, *Bacillus* species isolated from honeybee larvae showed similar antagonistic interactions *in vitro* (44). Moreover, *Bacillus* species from sources other than typical apiarian environments, or with unreported origins, also showed inhibition against *P. larvae in vitro*. These included *B. licheniformis*, *B. megaterium*, and *B. thuringiensis* subsp*. entomoidus* (7, 21). *B. amyloliquefaciens* LBM 5006, isolated from soil, exhibited antagonistic activity against *P. larvae* (20). These results harmonize with our findings, suggesting that *Bacillus s*pecies can suppress *P. larvae* strains *in vitro*. However, in this study, not all the bacterial isolates were evaluated against all *P. larvae* strains. Although the screening step allowed promising bacterial isolates to be selected for further evaluation, the incomplete combination of bacterial isolates and *P. larvae* strains limits our ability to determine the breadth and strain-specificity of their antagonistic activity. To better characterize the consistency and spectrum of inhibitory activity, future studies should evaluate a broader range of bacterial isolates against multiple genetically diverse *P. larvae* strains. We also tested a *Lactobacillus* strain from the NCIMB GenBank, originally isolated from bee guts, and this strain inhibited all the tested *P. larvae* strains (**Figure 1**). Prior findings reported similar results: *Lactiplantibacillus plantarum* strains (formerly *Lactobacillus plantarum*) exhibited antagonist activity against *P. larvae* (45). Likewise, two *Lactobacillus* phylotypes (Hma11 and Biut2) demonstrated strong inhibitory effects on the *in vitro* growth of *P. larvae* (6). Other lactic acid bacteria from different sources, such as fermented food products, can also inhibit *P. larvae* (46). Our results also show that this inhibitory activity may be specific to *P. larvae* because the microbiome of adult bees was not altered by the addition of the *Bacillus* lysate. This assertion deserves further attention as *Bacillus* species are generally known as producers of antimicrobials (47). It is interesting that this genus is regularly found in honeybees and does not seem to have large effects on other bee gut commensals.

Our results of ASx lysate inhibiting *P. larvae* suggested that the broadest-acting antimicrobial compounds have a molecular weight under 50 kDa and above 30 kDa (**Figure 2**; **Supplementary Figure S2**; **Table 1**). There are only a few reports in the literature on apiary-sourced or other *Bacillus*-associated proteins and/or bacteriocins tested against *P. larvae*. One study reported that *B. subtilis* isolated from the honeybee gut and honey produces an antimicrobial compound, surfactin, which inhibited *P. larvae in vitro* (18). Similarly, bacteriocin-like inhibitory substances (BLIS) obtained from *Bacillus cereus* isolated from honey have high inhibitory effects on *P. larvae* growth, and were also heat-stable at 70°C (17). Laterosporulin – a bacteriocin, an antimicrobial protein fraction, produced by a honeybee-derived strain of *Brevibacillus laterosporus* (formerly *Bacillus laterosporus*), successfully inhibited *P. larvae* in *in vitro* experiments (48). Our findings align with those of Benitez et al. (20), who reported that iturin produced by the soil-derived *B. amyloliquefaciens* strain LBM 5006 inhibited *P. larvae*. This antimicrobial peptide exhibited thermal stability up to 80°C and was susceptible to proteinase K. Furthermore, Cherif et al. (21) identified entomocin 110, a bacteriocin from *B. thuringiensis* subsp*. entomoidus* that mirrors our findings; this peptide inhibited *P. larvae in vitro*, was heat-stable, and its inhibitory activity was completely lost after treatment with proteinase K, indicating its proteinaceous nature. However, in our study, we used only the ASx lysate with a molecular weight ranging between 30–50 kDa; the active antimicrobial component(s) or metabolite(s) responsible for the inhibition, as well as their concentration used, were lacking. Another limitation is that the lysates (protein fractions) were separated using MWCO filtration, and we did not perform an independent molecular weight validation such as SDS-PAGE. The 30–50 kDa lysate fraction may contain proteins outside this molecular-weight range. Therefore, further protein characterization experiments are required to identify the active antimicrobial component(s) or metabolite(s).

Although there are no reports of *B. licheniformis*-associated proteins tested against *P. larvae*, this strain is considered a promising probiotic for apiculture because it can enhance honeybee immunity and protect against pathogens. It has been reported that the supplementation of the normal bee diet with *B. licheniformis* and other beneficial bacteria significantly reduced the mortality of AFB-infected larvae (11), which has been linked to increased survival rates in honeybee larvae. Our study also showed similar results (**Figure 4**); however, the only difference was that the study by Al-Ghamdi et al. (11) used *B. licheniformis* cell cultures, whereas we used the lysate (50–30 kDa) to feed larvae. It is possible that antimicrobial peptides (e.g., bacteriocins) associated with this strain played a role in the mortality reduction of the AFB-infected larvae in the study conducted by Al-Ghamdi et al. (11). However, this is just a presumption; to uncover the underlying mechanisms leading to the reduction in mortality among infected larvae in such infection studies, further investigation is necessary.

*B. licheniformis* has been reported to have significant probiotic potential in animal and human health in terms of modulation of the intestinal microbiota, antimicrobial activity, growth promotion, anti-inflammatory and immunostimulatory effects, promotion of the regulation of the lipid profile, increase of neurotransmitters, and stress reduction (49). Lipopeptides synthesized by the *B. licheniformis* B6 strain have potential antibacterial and anti-biofilm activity against pathogenic bacteria of health importance, suggesting it as a potential biocontrol alternative (50). This paper also mentioned that 70% of the reviewed articles reported *B. licheniformis* as a probiotic, out of which 75% used it in animal trials (such as on birds, including chickens, marine animals, and rodents) or in *in vivo* models. Additionally, *B. licheniformis* has proven to be a safe probiotic for consumption, with the ability to resist the conditions of the entire gastrointestinal system (49). Since *B. licheniformis* has proved itself safe and has already been used in the veterinary industry, it could also be used in apiculture for AFB, as it has shown positive results against *P. larvae* in honeybee larvae.

A review paper reported that *B. licheniformis* can produce various bacteriocins ranging in molecular weight from 1.4 to 55 kDa (51). A 30.7 kDa, YbdN protein extracted from *B. licheniformis* showed antibacterial activity against methicillin-resistant *Staphylococcus aureus*, vancomycin-resistant Enterococci, and *Listeria monocytogenes* (52). Likewise, antimicrobial compounds named F4, F5, and F6 with molecular mass less than 45 kDa, produced by *B. licheniformis* BFP011, showed resistance to pronase and high temperatures (100°C and 121°C for 15 minutes) and were able to inhibit the growth of gram-negative bacteria *Escherichia coli* and *Salmonella typhi* (53). Since *B. licheniformis* possesses remarkable plasticity as a secretion vehicle, the compounds it secretes depend on the growth medium composition, growth period, environmental conditions, and the specific strain (51).

Apart from *B. licheniformis*, we also tested cell cultures of 2D *Bacillus* and NCIMB Lacto in the laboratory-reared larvae against *P. larvae*. Our results showed that cell cultures of 2D *Bacillus*, supplemented with the larval diet, significantly improved larval survival, indicating the potential of this strain against AFB infections. *B. zhangzhouensis* was first proposed as a new *Bacillus* strain in 2016, isolated from aquaculture water from Zhangzhou city in China (54). Later, it was reported to be isolated from honey samples (55–57), bee pollen and bread (58), from seaweed (59), and recently from plants (60). Some of these isolates produce metabolites with growth inhibition potential against human and animal pathogens, including *Staphylococcus aureus, S. epidermidis, Candida albicans, Listeria monocytogenes* (56), and *Escherichia coli, Pseudomonas aeruginosa* (58). It has also been reported that a phthalate derivative extracted from an epibiotic bacterium, *B. zhangzhouensis* SK4, isolated from seaweeds, showed significant suppression of QS-regulated virulence factors of *P. aeruginosa* MCC 3457, which could deal with the multidrug resistance phenomenon (59). Recently, it has also been reported that *B. zhangzhouensis*, isolated from plants, produces secondary metabolites with antifungal and growth-regulating properties in plants (60). Although ASx lysate and 2D *Bacillus* improved the survival of larvae challenged with *P. larvae* strain 55 and *P. larvae* Y-3650 (belonging to ERIC group I), our study lacks *in vivo* testing of other ERIC groups. Future work should investigate strains from ERIC group II, as this genotype is the most virulent and is frequently isolated in Europe (61–63).

The NCIMB Lacto fed alone had no negative impacts on larval growth; however, larval survival significantly decreased when it was co-inoculated in the diet with *P. larvae*. Our findings did not match our own *in vitro* results and are contrary to the reports that demonstrated that the addition of different *Lactobacillus* species to the larval food improved honeybee larval survival during *P. larvae* infection (6, 64). These discrepancies might be explained by the fact that Forsgren et al. (6) and Daisley et al. (64) administered a mixture of lactic acid bacteria (LAB), including different *Lactobacillus* species, while we administered only a single *Lactobacillus* strain. Additionally, in the study by Daisley et al. (64), *Lactobacilli* strains were delivered through a nutrient biopatty instead of the liquid larval feed. This might be an additional reason why the supplemented NCIMB Lacto, in combination with *P. larvae* Y-3650 in the diet, failed to demonstrate growth suppression of *P. larvae*, contradicting the *in vitro* assay results. We did observe a trend towards increased survival, but the increase was not significant (*P*=0.98).

Honeybees are characterized by a uniquely conserved gut microbial community consisting of 5–8 bacterial phyla that support diverse host functions from metabolic regulation, cognitive enhancement, nutrient processing, to priming the host immune system (65–67). Gut symbionts colonize the hindgut in a successive order within the first 4–8 days post-NEW eclosion. If the microbial community abundance fluctuates across the insect’s lifespan, disruptions such as species loss or dysbiosis can result (68). Therefore, any treatment strategies against bacterial pathogens should consider the impact on the gut microbiome. Previous studies on the honeybee gut microbiome establishment reported that NEWs acquire their characteristic gut bacteria through exposure to hive components, horizontal transmission from hive mates, and coprophagia during early adult development (69, 70). As traditional *in vivo* hoarding cages lack these natural transmission routes, brood comb from the hive was provided to each cage to facilitate microbiome establishment in the absence of the hive environment. That this approach succeeded is evident in the resulting communities: caged bees developed the *Lactobacillus-*dominated gut characteristic of adult workers (the dominant genus in 38 of 44 samples, confirming that the provided comb was sufficient to seed the core microbiome without access to hive or hive-reared adult workers. Notably, *Bifidobacterium* (phylum Actinobacteria), a recognized member of this conserved core, was not detected in any sample in the present study, nor was any other *Actinobacterium*; this uniform, phylum-wide absence is characteristic of the amplification and extraction biases known to under-recover high-GC Actinobacteria in 16S rRNA surveys (71, 72).

To assess the impact of treatment and early exposure to ASx lysate on the bee gut microbiome, gut microbial communities were characterized by 16S rRNA gene sequencing and compared with those of bees treated with commonly administered antibiotics, Terra-Pro and tetracycline. We found that the survival of *Bacillus*-treated bees was lower than that of the sugar-syrup-fed (control) bees, but not different from that of the Terra-Pro-treated bees, the antibiotic growers use to treat AFB in the field. Beta diversity analysis of the relative abundance using Bray-Curtis dissimilarity indicated that ASx lysate treatment had minimal impact on microbiome composition relative to untreated controls, in contrast to the antibiotic treatments (**Figure 7**). Critically, ASx lysate produced no significant abundance changes in core microbiome species by either method and remained closest to untreated controls by Jaccard distance (0.38), whereas the two antibiotic treatments were most similar to one another (0.28). These results suggested that ASx lysate may offer a targeted approach that minimizes disruption to the beneficial core microbiome.

## 5 Conclusion

This study demonstrated that honeybee hive-associated *Bacillus* species have promising biocontrol potential against *P. larvae*, the causative agent of AFB. Several *Bacillus* isolates exhibited inhibitory activity against *P. larvae* strains *in vitro*, and selected *Bacillus* culture and lysate improved honeybee larval survival during pathogen infection. The antimicrobial activity of ASx lysate was associated with heat-stable proteins in the 30-50 kDa range and showed broad inhibitory activity against the tested *P. larvae* strains. Importantly, ASx lysate did not significantly affect adult bee survival compared with Terra-Pro and caused minimal effects on the gut microbiome. Overall, these findings support that hive-associated *Bacillus* species, particularly *B. licheniformis*, have promising biocontrol potential and represent candidates for the development of biological approaches to AFB management, providing a foundation for further evaluation under colony-level conditions.

## Data Availability

The datasets presented in this study can be found in online repositories. The names of the repository/repositories and accession number(s) can be found in the article/**Supplementary Material**.

## Supplementary material

Supplementary Figure 1 Representative plate pictures showing the effects of the tested strains against the *P. larvae* strains.

Supplementary Figure 2 *B. licheniformis* (ONT-R2-ASx) lysate fractions spotted on *P. larvae* lawns, in clockwise direction starting top left (A) Less than 0.22µM–greater than 100 kDa; (B) Less than 100 kDa–greater than 50 kDa; (C) Less than 50 kDa–greater than 30 kDa; (D) Less than 30 kDa. Two dilutions of each lysate size were spotted: top row – (10^0^) not diluted lysate; and bottom row–(10^-1^) 1:10 diluted lysate. Each lysate size and its ten-fold (1:10) dilutions were tested in triplicate (n=3).

Supplementary Figure 3 Representative plates of spot plate assay of heat-treated ASx lysate on the lawns of *P. larvae* strains. The lysate was serially diluted (10^0^ to 10^-3^), heat-treated, and spotted onto the lawns.
